# Editorial: Worsening heart failure: challenges, treatments, future perspectives

**DOI:** 10.3389/fcvm.2025.1578532

**Published:** 2025-04-03

**Authors:** Pietro Scicchitano, Anna Livrieri, Francesco Massari, Massimo Iacoviello

**Affiliations:** ^1^ Cardiology Section, Hospital “F. Perinei” ASL BA, Altamura, Italy; ^2^ Department of Medical and Surgical Sciences, University of Foggia, Foggia, Italy

**Keywords:** worsening heart failure, heart failure, epidemiology, treatments, physiopathogenesis

**Editorial on the Research Topic**
Worsening heart failure: challenges, treatments, future perspectives

Heart failure (HF) remains a major global health challenge, with rising prevalence and significant implications for morbidity and mortality (1). The clinical course of HF is characterized by the decline in clinical status of the patients till the occurrence of the final outcome (2). Each recurrence reduces the full-recovery of the patient and worsens his/her functional status.

Escalating signs and symptoms of HF in patients with chronic HF despite previously stable therapy define Worsening Heart Failure (WHF) status which recently emerges as the most intriguing clinical condition to be firmly addressed for preventing the worst outcome of HF (3). This implies the use of e.v. diuretics and the need for possible hospitalization and/or urgent outpatient evaluation.

The management of WHF is challenging as no definite therapy is able to counteract the outcome of patients who suffered for it. Despite the clinical benefit from the use of Empagliflozin in patients who are hospitalized for acute heart failure, the EMPagliflozin 10 mg compared with placebo, initiated in patients hospitalized for acUte heart faiLure who have been StabilisEd (EMPULSE) study did not demonstrate a definitely reduction in hospitalization for HF, cardiovascular death, and total HF hospitalizations (4).

Indeed, the use of Vericiguat—an oral soluble guanylate cyclase stimulator—in the setting of WHF effectively reduced the composite outcome of death from cardiovascular causes or first hospitalization for heart failure in the Vericiguat Global Study in Subjects with Heart Failure with Reduced Ejection Fraction (VICTORIA) (5).

Therefore, the need for implementing the knowledge about the mechanisms of WHF in order to provide more efficient treatments for counteracting its degenerative impact on the prognosis of patients is of utmost importance in cardiovascular medicine.

This special issue entitled “*Worsening Heart Failure: Challenges, Treatments, Future Perspectives”* seeks to explore the complexities of HF progression, emerging treatments, and the future landscape of HF management.

In this context, a useful insight was from Lopez-Vazquez et al. who explored the pathogenetic role of mitochondrial pyruvate carrier (MPC) expression in the landscape of heart failure with reduced ejection fraction (HFrEF) both in ischemic and non-ischemic subtypes. Specifically, reduced mRNA expression and protein concentration of MPC-1 and -2 in patients with both ischemic and non-ischemic HFrEF let supposed a possible role of abnormalities in this carrier in the pathogenesis of failure of cardiac muscular cells.

The landscape of pathogenesis of alterations in cardiac function and structure—which may lead to clinical destabilization and WHF occurrence—might be enriched by newer discoveries thanking to gene analysis. Revathi Venkateswaran et al. found a race-specific pattern in SNPs: seven independent loci related to human plasma metabolites were related to death in Black/African race patients, while no one was in White/European race patients. These genes might be implicated in further mechanisms able to explain the alterations in metabolic and energetic pathways at the origin of cardiac destabilization.

Nevertheless, the early identification of early signs and symptoms of WHF is a challenging issue. Gheorghiade et al. (6) previously observed that the diagnostic value of clinical markers of congestion is questionable: sensitivity, speciﬁcity, positive and negative predictive values are almost low and poorly related to the identification of early stages of decompensation. The combination of them into a multiparametric approach might be a correct solution for this issue (7, 8): by combining parameters clinicians might increase their discrimination skills, early identify those patients prone to decompensation, and intervene with effective therapeutic strategies for reducing worse outcomes. Hyun et al. highlights the utility of lung ultrasound in predicting respiratory complications in HF patients requiring mechanical ventilation, emphasizing the role of imaging in critical care management. Lung ultrasound would improve the recognition of patients who might benefit from weaning or those who should continue mechanical ventilation, thus ameliorating identification of the transition to better or worse outcomes, respectively (Hyun et al.).

As previously outlined, one of the most challenging issue is the need for more therapies and clinical strategies for counteracting WHR. Lu et al. managed an interesting overview about the use of Dengzhan Shengmai capsule, a traditional Chinese medicine, in patients with chronic heart failure. This article provided a comprehensive evaluation of its potential as a complementary therapy in the management of chronic HF. Through rigorous analysis of clinical data, the review offers insights into its efficacy in improving cardiac function and its safety profile, contributing to the growing body of evidence on integrative HF therapies.

Indeed, the need for reducing the occurrence of exacerbation of chronic HF and its destabilization into the WHF condition passes through the controlling of comorbidities which might impact on structure and function of the ventricles. Specifically, atrial fibrillation (AF) constitutes a determinant in the pathogenesis of heart failure decompensation (9, 10). AF and heart failure with preserved ejection fraction (HFpEF) are linked each other and promote the occurrence of WHR (9, 10). Strategies for weaning this interdependence should also focus on the control of the arrhythmia and management of its chaotic origin. Bai et al. the impact of the two main strategies—rate vs. rhythm-control in AF—on worsening HFpEF and structure cardiac remodelling. Specifically, they found that a rhythm-control management might reverse the structural remodelling of the left atrium and improve the clinical course of the diseases as compared to rate-control.

The inclusion of these diverse studies underscores the multifaceted nature of WHF and the need for innovative approaches in its management. While guideline-directed medical therapy (GDMT) remains the cornerstone of HF care, exploring novel therapies, genetic influences, mitochondrial function, imaging modalities, and electrophysiological strategies can enhance patient outcomes.

We hope that this special issue serves as a valuable resource for clinicians, researchers, and healthcare providers committed to improving the lives of individuals affected by worsening heart failure.
